# Relativistic Effects on NMR Parameters of Halogen-Bonded Complexes

**DOI:** 10.3390/molecules24234399

**Published:** 2019-12-02

**Authors:** Ibon Alkorta, José Elguero, Manuel Yáñez, Otilia Mó, M. Merced Montero-Campillo

**Affiliations:** 1 Instituto de Química Médica, IQM-CSIC. Juan de la Cierva, 3, 28006 Madrid, Spain; iqmbe17@iqm.csic.es; 2 Departamento de Química, Módulo 13, Facultad de Ciencias and Institute of Advanced Chemical Sciences (IadChem), Universidad Autónoma de Madrid, Campus de Excelencia UAM-CSIC, Cantoblanco, 28049 Madrid, Spain; manuel.yanez@uam.es (M.Y.); otilia.mo@uam.es (O.M.)

**Keywords:** halogen bonding, astatine, relativistic effects, NMR, absolute shieldings, nuclear quadrupolar coupling constants

## Abstract

Relativistic effects are found to be important for the estimation of NMR parameters in halogen-bonded complexes, mainly when they involve the heavier elements, iodine and astatine. A detailed study of 60 binary complexes formed between dihalogen molecules (XY with X, Y = F, Cl, Br, I and At) and four Lewis bases (NH_3_, H_2_O, PH_3_ and SH_2_) was carried out at the MP2/aug-cc-pVTZ/aug-cc-pVTZ-PP computational level to show the extent of these effects. The NMR parameters (shielding and nuclear quadrupolar coupling constants) were computed using the relativistic Hamiltonian ZORA and compared to the values obtained with a non-relativistic Hamiltonian. The results show a mixture of the importance of the relativistic corrections as both the size of the halogen atom and the proximity of this atom to the basic site of the Lewis base increase.

## 1. Introduction

Second in importance after hydrogen bonds, halogen bonds (XB) are widely present in many fields such as crystal engineering, biological systems, and the design of new materials, amongst others. It is worth citing here the IUPAC definition of the halogen bond: “A halogen bond occurs when there is evidence of a net attractive interaction between an electrophilic region associated with a halogen atom in a molecular entity and a nucleophilic region in another, or the same, molecular entity” [1]. The properties of this bond have been reviewed in two recent books on this topic [2,3].

A key feature of halogen molecules is the polar flattening of the electron density [4,5,6], also known as σ-hole [6,7,8]. This phenomenon is responsible for the directionality of the halogen bond when halogens interact with a Lewis base, a property with an enormous influence on the strength of non-covalent bonds. When combined with other bonds, the same or different, positive and negative cooperativity effects are observed [9,10,11,12].

In the gas phase, the experimental evidence of halogen bonds and their properties is usually obtained by microwave spectroscopy. A suitable example is the values reported by Legon et al. by comparing halogen bonds with HBs [13]. In the solid state, this information is obtained by means of nuclear quadrupole resonance techniques, as in the study carried out by Bryce et al. [14,15,16]. Moreover, relationships between the dissociation energies, De, and the nucleophilicity and electrophilicity in XB have been reported [17,18]. Halogen bonds with typical Lewis bases such as phosphines, H_2_XP:Cl_2_, show traditional and shared halogen bonds [19,20,21], with the strength of these two kinds of complexes depending on the donor ability of the phosphine.

It is well known that relativistic effects become more and more important when the size of the system increases.

Complexes between halogens and small Lewis donors were usually the simplest models for the study of the halogen bonds, but little work has been done on the relativistic effects on this kind of bond. This can be justified by taking into account that relativistic effects become particularly important when the size of the atoms involved increases. However, in a very interesting paper on chemical shieldings and spin–spin coupling constants, P. Pyykkö clearly showed that relativistic effects were “more common than you thought” [22]; but, for sure, they should be non-negligible when dealing with iodine or astatine derivatives. Even though if it was not so common to introduce such effects in the theoretical treatment, [23,24], a recent paper on halogen bonds involving astatine clearly shows the important role these effects may have in the description of the bonds we are dealing with [25].

**Figure 1:**
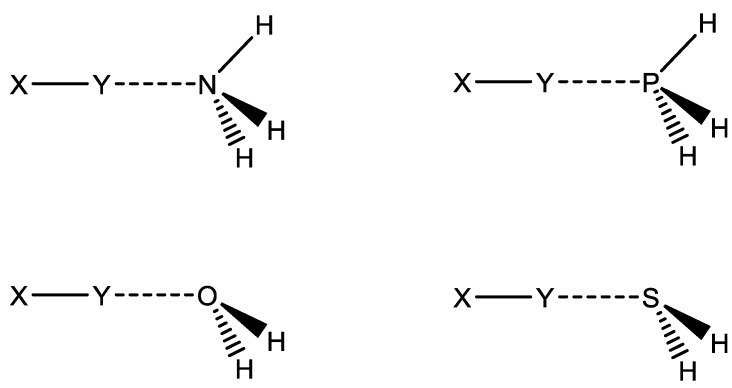
Schematic representation of the complexes studied. (X, Y = F, Cl, Br, I, and At).

In this article, the complexes between all the dihalogen molecules including F, Cl, Br, I, and At and four Lewis bases (NH_3_, PH_3_, H_2_O, and SH_2_) have been studied (Figure 1). Their dissociation energies, geometries, and NMR properties (chemical shielding and nuclear quadrupole coupling constants) have been obtained and analyzed. Calculations were done using a non-relativistic and a relativistic ZORA Hamiltonian to assess the importance of relativistic effects.

## 2. Computational Methods

The geometry of the complexes was optimized at the MP2 computational level [26] with the aug-cc-pVTZ basis set [27,28]. For iodine and astatine, the effective core potential basis set (ECP) and the aug-cc-pVTZ-PP basis set were used [29]. For complexes involving NH_3_ and PH_3_, the geometry optimization was done assuming a *C_3v_* symmetry and a *C_s_* symmetry for those involving H_2_O and SH_2_. Vibrational frequency calculations at the same computational level were carried out to confirm that the obtained structures correspond to local minima of the potential energy surface. These calculations were performed with the Gaussian-16 program [30].

In order to provide some insight on the characteristics of the halogen bonds investigated, we decided to use the energy decomposition analysis (EDA) [31] of the interaction energy (Equation (1)), which was carried out with the ADF-2017 program [32] to obtain information of the different energy contributions:

E_int_ = E_Pauli_ + V_elst_ + E_orb_ (1)

The Pauli repulsion is associated with the destabilizing interactions between occupied orbitals and is responsible for any steric repulsion. The V_elst_ term corresponds to the classical electrostatic interaction between the two molecules in the geometry of the complexes. The orbital energy, E_orb_, accounts for the charge transfer and polarization.

Relativistic corrected NMR chemical shieldings and nuclear quadrupole coupling constants (NQCC) were obtained using the full electron QZ4P basis [33], together with the PB86 functional [34,35,36], and the relativistic ZORA spin-orbit Hamiltonian [37,38]. In addition, non-relativistic calculations were performed with the same functional and basis set to check the influence of relativistic corrections. For the calculation of the NQCC parameters of astatine, a recently proposed nuclear quadrupole moment of −0.42 barn, was used [39]. These calculations were performed using the ADF-2017 program [32].

The molecular electrostatic potential (MEP) was calculated within the Gaussian-16 facilities and analyzed on the 0.001 au electron density isosurface with the Multiwfn program [40]. The corresponding figures were generated with the JMol program [41]. For the calculation of the MEP no relativistic effects were included, assuming that they will have a small effect on the value of the potential.

## 3. Results and Discussion

### 3.1. Isolated Monomers

Prior to the study of the halogen-bonded complexes, an exploration of the properties of the halogen molecules was carried out. The calculated and experimental interatomic distances of the isolated XY molecules are gathered in Table 1. Experimental geometries are available for all the diatomic molecules save for the At derivatives. The calculated values are in good agreement with the experimental ones, the largest error being 0.01 Å. The calculated and experimental distances show an almost perfect linear correlation (R^2^ = 0.9997, *n* = 10).

**Figure 2:**
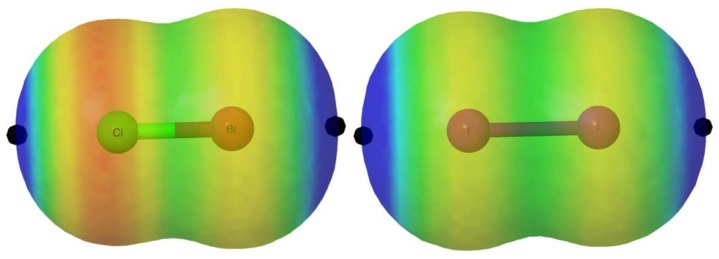
Molecular electrostatic potential (MEP) on the 0.001 a.u. electron density isosurface of the ClBr and I_2_ molecules. The location of the σ-hole is indicated with a black dot. The color code range between values > 0.015 au in blue and <−0.010 au in red.

The MEP of these molecules, in agreement with the expected polar flattering already explained in the introduction, presents two σ-holes along the X-Y bond associated with the two atoms (Table 1). In Figure 2, we illustrate the MEP for the particular cases of ClBr and I_2_ as representative examples, for which the halo of the lone pairs is easily visualized in red color whereas the σ-hole is markedly blue.

These holes are maxima of the MEP and exhibit positive values. It is important to emphasize, however, that, as already reported in the literature [42], σ-holes do not always present positive MEP values. Indeed, within the systems studied in this work, some of the F derivatives show local maxima with negative values of the MEP. In all cases, the largest σ-hole is associated with the heavier of the two atoms (Y from now on) because of its larger polarizability. For a given Y atom, the value of the σ-hole decreases as the size of X increases, which is associated with a smaller electron withdrawing ability of X. In a series of compounds with the same Y, the changes in the values of the σ-holes follow the changes in the atomic radius of the X atom. Indeed, for Y = At, the largest gap in the value of the σ-hole (0.04 a.u.) is observed on going from F to Cl, with an increase in the atomic radius of 50 pm [43]. Going from Cl to Br, the decrease found in the σ-hole was reduced to 0.012 a.u., with an increase in the atomic radius of only 12 pm. Going from Br to I, the gap between their atomic radii slightly increased (25 pm) and, concomitantly, the gap in the values of the σ-hole (0.017 a.u.) also increased. This finding confirms that the size effects along the periodic table are particularly important when going from the first to the second period. For a given X, the σ-hole increased as the size of the Y atom did, following the increase of the polarizability of the latter. For instance, in the series F_2_, FCl, FBr, FI, FAt, the values of the σ-hole increased from 0.0211 a.u. to 0.1310 a.u. (see Table 1).

The nuclear quadrupole coupling constants (NQCC) of the isolated dihalogen molecules are gathered in Table 2. The calculated parameters are in good agreement with the experimental ones.

**Figure 3:**
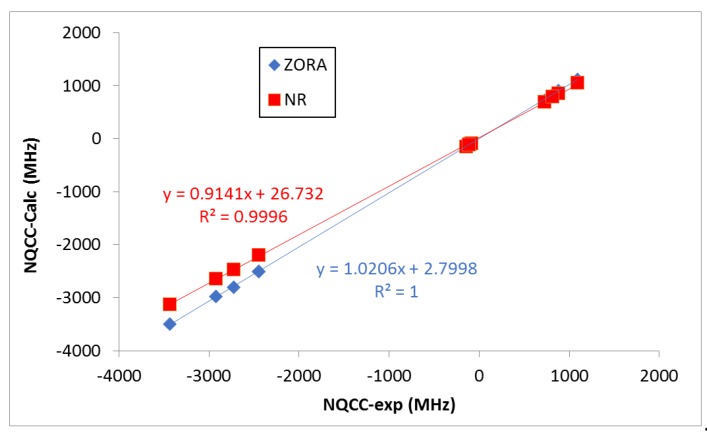
Experimental vs. calculated NQCC parameters (MHz). The fitted linear relationships between the experimental and calculated values are shown.

It should be noted, however, that the deviations of the NR calculated values with respect to the experimental ones are larger than those obtained when relativistic effects are accounted for (ZORA). As expected, the heavier the halogen atom, the larger the effect. This is seen more clearly when looking at the correlations shown in Figure 3. Indeed, ZORA results present a slightly better R^2^ value, a slope closer to 1.0, and an intercept value closer to 0.0 (Figure 3). Negative NQCC values were obtained for the ^35^Cl, ^127^I and ^210^At nucleus, while positive ones were obtained for the ^79^Br nucleus (as observed in experiments). The positive and negative values of NQCC are associated with the effective shape (prolate and oblate, respectively) of the equivalent ellipsoid of the nuclear charge distribution [46,47]. The absolute average values for each nucleus increased steadily with its size. For a given X (or Y), the NQCC values decreased in absolute value as the size of Y (or X) does.

### 3.2. Dissociation Energies

Once the properties of the XY molecules were analyzed, we proceeded to study their complexes. The dissociation energies (De) of the XY: Base binary complexes are reported in Table 3. The values ranged between 5.6 kJ·mol^−1^ for the F_2_:PH_3_ and F_2_:SH_2_ complexes and 97.7 kJ·mol^−1^ for the FAt: NH_3_ complex. Among the trends observed in Table 3, it is interesting to notice that for a given Y atom and base, De was smaller as the size of X increased. The largest difference between two consecutive Xs is between F and Cl. Concerning the base, the general trend is NH_3_ > PH_3_ > SH_2_ > H_2_O with only two exceptions: The F_2_:OH_2_ complex that is slightly more stable than the corresponding PH_3_ and SH_2_ ones, and FCl:PH_3_ that is also slightly more stable than FCl: NH_3_.

The dissociation energies listed in Table 3 can be fitted using Equation (2) proposed by Legon and Millen that relates these energies with a nucleophilic parameter characterizing the bases, *N_b_*, and an electrophilic parameter characterizing the Lewis acids, *E_a_* [17,48,49].

(2) $$D_{e}=c\cdot N_{b}\cdot E_{a}$$

where the constant c has a value of 1.00 kJ·mol^−1^ to preserve the units of the equation.

In the present case, we have four *N_b_* values and 15 *E_a_* values to be fitted, so a total of 60 possible combinations. The simultaneous fitting of the nucleophilicities and electrophilicities is done by means of Equation (3).

(3) $$D_{e}=c\cdot \left(\overset{4}{\underset{i=1}{\sum }}x_{i\;}\cdot \;N_{bi}\right)\cdot \left(\overset{15}{\underset{j=1}{\sum }}x_{j\;}\cdot \;E_{aj}\right)$$

where the values of *x_i_* and *x_j_* are 1.0 when the corresponding Lewis base or Lewis acid is present in the complex, and 0.0 if it is absent.

The fitted values for each base and acid are given in Table 4. The fitted equation presents a R^2^ value of 0.988 and an average unsigned error of 1.7 kJ·mol^−1^, the largest error (7.8 kJ·mol^−1^) being found for the FCl:PH_3_ complex. It is known that this complex has a significant ion-pair character F^−^···ClPH_3_^+^ and, as a consequence, an enhanced De [50], which explains this large deviation. Removing the two worst-fitted values (FCl:PH_3_ and FBr:PH_3_), the correlation parameter improved significantly up to R^2^ = 0.994 with an average error of 1.3 kJ·mol^−1^.

The *N_b_* and *E_a_* values obtained for the set of compounds studied here are compared, in Table 4, with others reported in the literature. *N_b_* values in [17] are averaged among 250 complexes including hydrogen bonds, tetrel bonds, pnictogen bonds, chalcogen bonds, and halogen bonds. It is interesting to notice that PH_3_ and SH_2_ are stronger nucleophiles in halogen bonds than in the rest of the interactions studied in [17]. The same happened in the hydrogen bonds used to fit Equation (2) in [48]. In order to verify whether this increase in the *N_b_* values is due to the presence of the iodine and astatine derivatives not included in [17], we did a new fitting excluding the derivatives of these two elements. The new results of *N_b_* for PH_3_ and SH_2_ were indeed smaller (6.45 and 4.08, respectively), but the decrease is not significant and the same effect was also observed for the other bases, NH_3_ and H_2_O (new values 7.25 and 3.67, respectively) not affected by a significant change with respect to the values in [17]. With respect to the *E_a_* values for the dihalogen molecules, they are similar to those reported in [17] since both cases correspond to halogen bonds, though calculated at a slightly different computational level.

**Figure 4:**
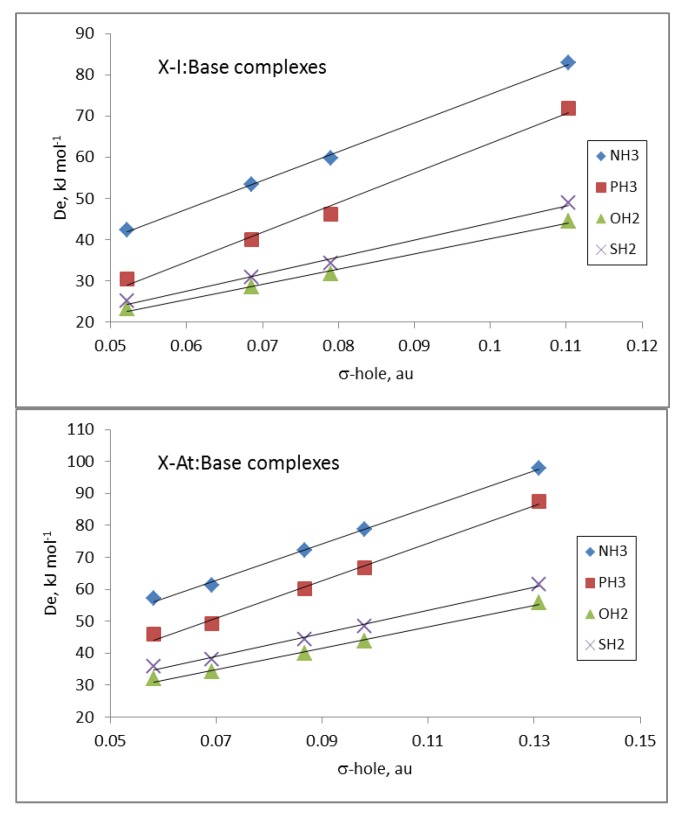
De vs. σ-hole in the X-I: Base (**up**) and X-At: Base (**down**) complexes as a function of the Base.

The electrostatic nature of these halogen-bonded complexes can be confirmed by comparing the *D_e_* values of all the complexes of a given base with the corresponding σ-hole values associated with the Y atom in the isolated dihalogen molecule (Table 1). Linear correlations between the *D_e_* and σ-hole values, with R^2^ between 0.89 and 0.92, were obtained for the complexes with each base. These results clearly improved if the complexes were separated in groups attending to the nature of the Y atom and the base involved in the interaction. Thus, in Figure 4, the relationships for the complexes with iodine and astatine are depicted, showing a linear behavior with R^2^ > 0.99. These results strongly indicate that other component in addition to electrostatics should be taken into account for a fine-tuning of the estimation of the De.

A more detailed analysis of interaction energy was carried out by means of the energy decomposition analysis (Table S1 of the Supporting Information). The results show that the two attractive terms (Pauli and orbital) are important in the stabilization of the complexes, being percentage contributions between 34% and 67% depending on the base and nature of the Y atom, with minimal influence of the X one. The exception to this trend is the FCl:PH_3_ complex, where the electrostatic contribution was only 22% and the orbital one 78%. In all the cases, for a given Y atom, the absolute value of the attractive contributions (Pauli, electrostatic and orbitals) decreased as the size of the X atom increased. This is due to the two facts; on the one hand, when X increased, the polarization of Y was smaller, and then the sigma-hole on Y was less deep; and on the other hand, the intermolecular distance increased. For a given X atom, the absolute value of the Pauli and electrostatic contributions increased as the size of Y increases. The FY:PH_3_ complexes are an exception to this rule.

### 3.3. Halogen Bond Distances

Table 5 collects the halogen bond distances for the whole set of 60 complexes, comparing the MP2/aug-cc-pVTZ values with the experimental ones available in the literature [13,46,47,48]. The halogen bond distance for the 16 halogen-bonded complexes experimentally known were reasonably well reproduced by our calculations (R^2^ = 0.98), especially considering that the experimental values included the ZPE effect on the geometry that is not included in the calculations of the optimized geometries. Thus, the calculated values were always shorter than experimental ones in average (0.14 Å).

The calculated halogen bond distances show that in all cases, for a given Y atom and the same base, the distance increased along with the X size because, as mentioned above, the σ-hole at the Y atom became shallower. In general, the distances for the same XY molecule followed the trend NH_3_ < H_2_O < PH_3_ < SH_2_, except in three cases: The F_2_: PH_3_ complex showed a slightly larger distance than the F_2_:SH_2_, and the FBr:PH_3_ and ClBr:PH_3_ distances were shorter than the corresponding one with H_2_O. Interestingly, as shown in Table 6, linear correlations R^2^ > 0.92 were found between the dissociation energies and the halogen bond distances for the complexes with same Y atom and base. The cases of XF and XCl are not included because the number of points is not sufficient.

### 3.4. NMR Absolute Shieldings

The chemical shieldings of the atom of the Lewis base directly involved in the halogen bond are reported in Table 7 and Table 8. They were smaller than those in the isolated bases, save in five cases where the relativistic correction was able to revert the trend, as is the case for complexes with FI and FAt.

**Figure 5:**
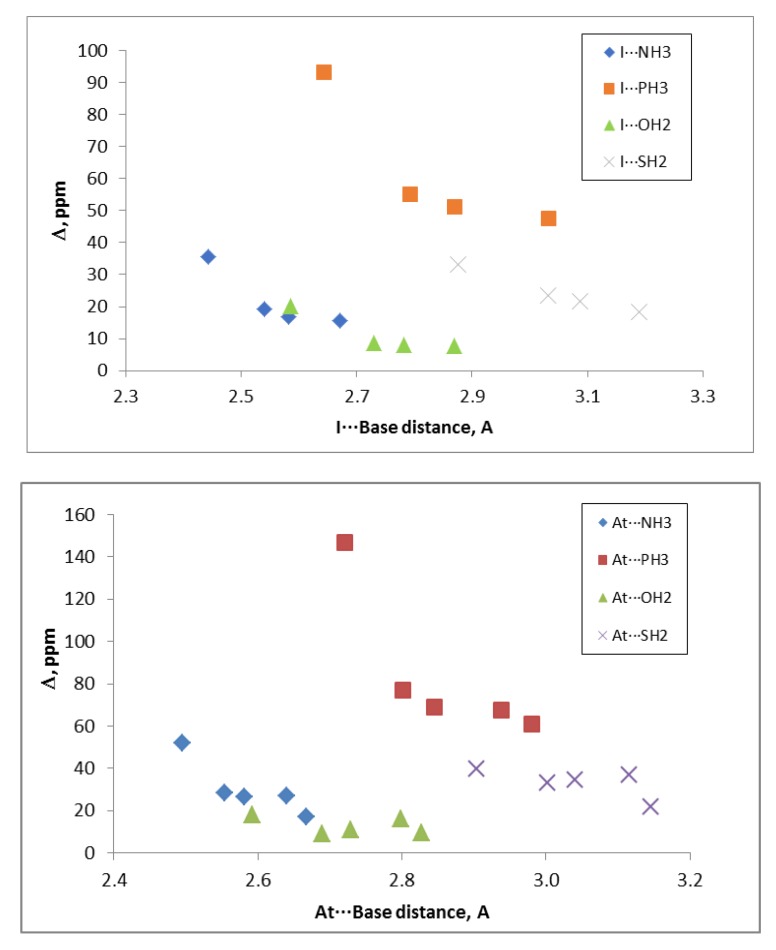
Relativistic effect on the chemical shielding, Δ, of the base on I: Base and At: Base interactions vs. the intermolecular distance.

The relativistic correction was always positive with values up to 147 ppm in the FAt:PH_3_ complex. Although no clear correlations were found between the intermolecular distances and the non-relativistic chemical shieldings, a clear dependence with the distance and the nuclei was observed for the values including relativistic corrections (see Figure 5).

### 3.5. NQCC

The NQCC values of the halogen atoms XY in the binary complexes calculated with the ZORA Hamiltonian are gathered in Table 9. Remember that ^19^F has no NQCC. Significant variations of this parameter were observed for both nuclei X and Y upon complexation. In the case of X, positive variations with respect to the corresponding value in the isolated XY molecules (see Table 2) were observed for the ^35^Cl, ^127^I, and ^210^At nuclei while negative variations were found for the ^79^Br one. Thus, the absolute values of the binary complexes were, from 7 to 831 MHz, smaller than the ones in the isolated XY molecules. For a given Y and base, the variation in absolute value followed the sequence Cl < Br < I < At. For a given XY molecule, the variation of the NQCC of X, with respect to the base, was larger in NH_3_ and PH_3_ than SH_2_, the complexes with H_2_O being the ones with the smallest variations.

In the case of Y, the variation was more negative (or less positive) as the size of the X atom increased for ^35^Cl and ^127^I, while for ^79^Br and ^210^At nuclei, the reverse trend was observed.

Recently, a relationship has been explored between the variation of the NQCC of the dihalogen molecules isolated and in complexes and the charge redistribution using the Townes–Dailey model [53]. Similar results have been obtained from the theoretical charge distribution obtained within the atoms in molecules methodology [54].

## 4. Conclusions

NMR and NQCC parameters, dissociation energies, and intermolecular distances were studied in detail for 60 halogen-bonded complexes between XY halogen molecules and a set of Lewis bases, namely NH_3_, PH_3_, SH_2_, and H_2_O, taking into account relativistic effects. Isolated halogen molecules exhibited negative NQCC values for ^35^Cl, ^127^I, and ^210^At nucleus, and positive ones for ^79^Br; the bigger the atom, the larger the absolute NQCC value. In the binary complexes XY: Base, the NQCCs calculated for X, including relativistic effects, present positive variations with respect to isolated XY molecule for ^35^Cl, ^127^I, and ^210^At, and negative for ^79^Br. This implies a reduction in absolute value of the NQCCs with respect to isolated halogens for all the nuclei, the variation following the trend Cl < Br < I < At. Among the Lewis bases, for a same XY molecule, NH_3_ and H_2_O led to the largest and shortest variations, respectively. On the other hand, the chemical shieldings of the atoms of the bases directly involved in the interaction were smaller than in the isolated bases, with some exceptions where the relativistic correction reverts the trend. This relativistic correction is always positive, reaching the highest value (147 ppm) for the FAt:PH_3_ complex.

Regarding the energy of the halogen-bonded complexes, molecular fluorine complexes show the smallest dissociation energies (5.6 kJ·mol^−1^ for both F_2_:PH_3_ and F_2_:SH_2_) and the FAt complexes the largest ones (97.7 kJ·mol^−1^ for FAt: NH_3_). Again, the general trend shows that NH_3_ leads to the strongest complexes and H_2_O to the weakest ones, with few exceptions. Dissociation energies fit well with nucleophilicity and electrophilicity parameters proposed by Legon (R^2^ = 0.994 if only two anomalous values are removed from the fitting). The importance of electrostatics is evidenced by the fact that dissociation energies and σ-hole MEP values of halogens are mostly linearly correlated (R^2^ = 0.89–0.92), but for complexes with iodine and astatine, this correlation is almost perfect (R^2^ > 0.99), indicating that not only electrostatic factors should be taken into account for smaller halogens.

## Figures and Tables

**Table 1:** Interatomic distances (Å) and σ-hole values (au) in the isolated X-Y dihalogen molecules.

	X-Y Distance		σ-Hole	
X-Y	Calculated	Experimental [44]	X	Y
F_2_	1.401	1.4119	0.0211	0.0211
FCl	1.638	1.6283	−0.0125 *	0.0762
Cl_2_	1.999	1.9879	0.0434	0.0434
FBr	1.758	1.7589	0.0162	0.0790
ClBr	2.138	2.1360	0.0320	0.0597
Br_2_	2.279	2.2811	0.0485	0.0485
FI	1.920	1.9098	−0.0354 *	0.1103
ClI	2.321	2.3209	0.0162	0.0790
BrI	2.465	2.4690	0.0320	0.0686
I_2_	2.663	2.6655	0.0521	0.0521
FAt	2.006		−0.0454 *	0.1310
ClAt	2.407		0.0063	0.0980
BrAt	2.550		0.0217	0.0867
IAt	2.749		0.0419	0.0692
At_2_	2.834		0.0582	0.0582

* These values correspond to local maxima along the F-Y bond.

**Table 2:** Experimental and calculated values of the nuclear quadrupole coupling constants (NQCC) (MHz) with and without the relativistic ZORA Hamiltonian of the isolated X-Y dihalogen molecules. NR stands for non-relativistic.

	X Atom				Y Atom			
X-Y	Nuclei	Exp. [45]	ZORA	NR	Nuclei	Exp. [45]	ZORA	NR
F_2_	^19^F	^a^			^19^F			
FCl	^19^F				^35^Cl	−145.87182	−146.45	−145.46
Cl_2._	^35^Cl	−115.0	−113.27	−112.42	^35^Cl	−115.0	−113.27	−112.42
FBr	^19^F				^79^Br	+1086.89197	1115.10	1060.86
ClBr	^35^Cl	−102.378	−103.50	−103.05	^79^Br	+875.078	903.353	857.40
Br_2_	^79^Br	+810.0	836.21	792.81	^79^Br	+810.0	836.21	792.80
FI	^19^F				^127^I	−3440.748	−3489.90	−3118.75
ClI	^35^Cl	−85.8	−87.2248	−88.17	^127^I	−2929.0	−2975.69	−2638.75
BrI	^79^Br	+722.0	722.50	690.58	^127^I	−2731.0	−2801.56	−2466.99
I_2_	^127^I	−2452.5837	−2509.26	−2195.70	^127^I	−2452.5837	−2509.23	−2195.66
FAt	^19^F				^210^At		−3553.36	−2788.80
ClAt	^35^Cl		−71.0026	−80.93	^210^At		−3105.21	−212.80
BrAt	^79^Br		600.699	639.03	^210^At		−3035.26	−2257.50
IAt	^127^I		−2172.51	−2049.92	^210^At		−2738.91	−2025.54
At_2_	^210^At		−2634.25	−1898.71	^210^At		−2634.25	−1898.71

^a 19^F has a nullvalue of the nuclear quadrupole moment and consequently the NQCC is always 0.0 MHz.

**Table 3:** Dissociation energies (De, kJ·mol^−1^) of the XY: Base complexes.

XY	Base = NH_3_	PH_3_	H_2_O	SH_2_
F_2_	8.5	5.6	6.0	5.6
FCl	49.5	50.9	23.4	25.8
Cl_2_	22.9	14.4	13.2	13.6
FBr	70.2	65.9	35.0	40.1
ClBr	42.2	31.5	22.2	24.0
Br_2_	36.0	26.0	19.4	21.3
FI	82.8	71.8	44.4	48.9
ClI	59.8	46.2	31.7	34.3
BrI	53.3	40.1	28.5	30.9
I_2_	42.4	30.4	23.2	25.1
FAt	97.7	87.4	55.8	61.6
ClAt	78.7	66.6	43.8	48.4
BrAt	72.2	60.0	40.0	44.4
IAt	61.3	49.2	34.2	37.9
At_2_	57.1	46.0	32.0	35.8

**Table 4:** Fitted *N_b_* (nucleophilicity) and *E_a_* (electrophilicity) values using the values gathered in Table 3 and Equation (3). The values described in the literature for some of these molecules are also included.

	*N_b_* Value	Ref. [17]	Ref. [48]
NH_3_	8.65	7.52	11.5
PH_3_	7.32	3.12	4.4
H_2_O	4.70	4.89	10.0
SH_2_	5.19	3.43	4.8
	*E_a_* **Value**		
F_2_	0.97	1.13	
FCl	5.89	5.18	
Cl_2_	2.46	2.71	
FBr	8.24	-	
ClBr	4.65	4.77	
Br_2_	3.96	4.40	
FI	9.61	-	
ClI	6.66	-	
BrI	5.91	-	
I_2_	4.67	-	
FAt	11.65	-	
ClAt	9.16	-	
BrAt	8.35	-	
IAt	7.03	-	
At_2_	6.58	-	

**Table 5:** Halogen bond distances (Å) for the XY: Base complexes.

	Calculated				Experimental			
XY	NH_3_	PH_3_	H_2_O	SH_2_	NH_3_	PH_3_	H_2_O	SH_2_
F_2_	2.594	3.048	2.649	3.021	2.708 [21]		2.719 [21]	3.200 [13]
FCl	2.233	2.659	2.517	2.721	2.370 [21]		2.611 [13]	2.857 [13]
Cl_2_	2.592	3.050	2.774	3.100	2.730 [21]	3.240 [13]		3.249 [13]
FBr	2.293	2.389	2.493	2.720				
ClBr	2.469	2.659	2.698	2.972	2.628 [13]			3.096 [13]
Br_2_	2.538	2.836	2.757	3.045	2.720 [21]			
FI	2.443	2.642	2.585	2.876				
ClI	2.540	2.792	2.730	3.032	2.711 [51]	2.963 [51]	2.828 [52]	3.154 [51]
BrI	2.582	2.868	2.781	3.087				
I_2_	2.672	3.033	2.869	3.190				
FAt	2.496	2.721	2.593	2.903				
ClAt	2.554	2.802	2.689	3.003				
BrAt	2.582	2.845	2.729	3.041				
IAt	2.640	2.939	2.799	3.116				
At_2_	2.667	2.981	2.828	3.146				

**Table 6:** R^2^ values for the linear correlations between De and the halogen bond distance.

Base	X-Br:Base	X-I:Base	X-At:Base
NH_3_	0.989	0.967	0.975
PH_3_	0.926	0.918	0.946
H_2_O	0.998	0.989	0.987
SH_2_	0.994	0.979	0.984

**Table 7:** Absolute chemical shielding (ppm) of the atom of the base involved in the interaction. The difference (**∆**) between the non-relativistic (NR) and relativistic (ZORA) results is also listed.

	NH_3_ Complexes (^15^N NMR)			PH_3_ Complexes (^31^P NMR)		
XY	NR	ZORA	Δ	NR	ZORA	Δ
-	259.67	260.59	0.92	569.22	580.5	11.28
F_2_	238.03	239.46	1.43	538.58	550.72	12.14
FCl	222.7	226.35	3.65	444.03	463.76	19.73
Cl_2_	230.4	232.41	2.01	531.28	544.73	13.45
FBr	229.76	245.21	15.45	454.34	500.82	46.48
ClBr	225.08	232.7	7.62	493.61	523.33	29.72
Br_2_	225.15	233.9	8.75	510.84	544.79	33.95
FI	237.04	272.69	35.65	465.54	558.92	93.38
ClI	227.13	246.29	19.16	494.14	549.38	55.24
BrI	224.83	241.62	16.79	504.27	555.62	51.35
I_2_	222.67	238.15	15.48	520.59	568.36	47.77
FAt	241.56	293.55	51.99	472.31	619.06	146.75
ClAt	230.24	258.86	28.62	496.22	573.18	76.96
BrAt	227.03	253.38	26.35	504.9	573.96	69.06
IAt	222.32	249.37	27.05	518.22	585.65	67.43
At_2_	220.25	237.28	17.03	525.75	586.92	61.17

**Table 8:** ^16^O and ^32^S absolute shieldings.

	H_2_O Complexes (^16^O NMR)			SH_2_ Complexes (^32^S NMR)		
XY	NR	ZORA	Δ	NR	ZORA	Δ
-	326.51	328.15	1.64	708.5	723.73	15.23
F_2_	311.27	312.88	1.61	660.08	674.88	14.80
FCl	302.28	304.79	2.51	633.48	649.49	16.01
Cl_2_	304.16	306.01	1.85	659.70	674.74	15.04
FBr	308.26	316.74	8.48	636.66	659.18	22.52
ClBr	300.21	303.96	3.75	646.51	664.01	17.50
Br_2_	299.46	303.58	4.12	647.36	663.65	16.29
FI	317.13	337.31	20.18	649.47	682.55	33.08
ClI	300.14	308.94	8.8	645.90	669.24	23.34
BrI	297.5	305.53	8.03	644.13	665.72	21.59
I_2_	294.91	302.81	7.9	642.76	661.24	18.48
FAt	322.85	341	18.15	654.03	693.74	39.71
ClAt	302.38	311.67	9.29	645.26	678.32	33.06
BrAt	297.8	308.72	10.92	641.84	676.2	34.36
IAt	291.66	307.79	16.13	636.52	673.34	36.82
At_2_	288.79	298.26	9.47	633.27	655.14	21.87

**Table:** (**a**)

XY	X Nuclei	Base = NH_3_	PH_3_	H_2_O	SH_2_
F_2_	^19^F	-	-	-	-
FCl	^19^F	-	-	-	-
Cl_2_	^35^Cl	−96.67	−101.60	−106.45	−103.33
FBr	^19^F	-	-	-	-
ClBr	^35^Cl	−80.73	−75.90	−94.06	−89.17
Br_2_	^79^Br	666.62	669.73	766.09	731.14
FI	^19^F	-	-	-	-
ClI	^35^Cl	−65.83	−62.74	−77.20	−73.07
BrI	^79^Br	550.33	539.92	643.91	611.72
I_2_	^127^I	−1978.37	−2027.60	−2275.95	−2182.00
FAt	^19^F	-	-	-	-
ClAt	^35^Cl	−53.71	−51.30	−61.91	−58.97
BrAt	^79^Br	453.45	439.72	524.63	500.39
IAt	^127^I	−1656.06	−1651.19	−1912.51	−1833.11
At_2_	^210^At	−2020.99	−2009.78	−2220.53	−1803.44

**Table:** (**b**)

XY	Y-Nuclei	Base = NH_3_	PH_3_	H_2_O	SH_2_
F_2_	^19^F	-	-	-	-
FCl	^35^Cl	−136.74	−104.16	−144.73	−136.22
Cl_2_	^35^Cl	−113.90	−111.54	−114.86	−111.80
FBr	^79^Br	1031.94	861.95	1093.30	1017.95
ClBr	^79^Br	898.39	832.30	914.18	877.07
Br_2_	^79^Br	841.11	799.32	850.08	818.11
FI	^127^I	−3292.39	−2920.15	−3453.39	−3239.75
ClI	^127^I	−2968.23	−2745.65	−3024.02	−2888.87
BrI	^127^I	−2823.84	−2641.50	−2859.06	−2738.31
I_2_	^127^I	−2583.75	−2457.53	−2582.64	−2490.00
FAt	^210^At	−3603.13	−3254.61	−3613.52	−3400.17
ClAt	^210^At	−3275.35	−3054.35	−3209.75	−3108.67
BrAt	^210^At	−3056.78	−2950.25	−3129.21	−3071.55
IAt	^210^At	−2977.79	−2799.42	−2883.22	−2768.93
At_2_	^210^At	−2921.46	−2688.23	−2748.77	−2588.70

## Supplementary Materials

The following are available online at https://www.mdpi.com/1420-3049/24/23/4399/s1: Information regarding coordinates and MP2 energies.
