# Supplementary material for: Relativistic Effects on NMR Parameters of Halogen-Bonded Complexes
Source: Molecules. 2019 Dec 2;24(23):4399. doi: 10.3390/molecules24234399 (PMC6930553; doi:10.3390/molecules24234399)
Supplement: Supplementary file 1 [file molecules-24-04399-s001.pdf]

Supporting Information

Relativistic Effects on NMR Parameters of Halogen Bonded Complexes

Ibon Alkorta [1,*], José Elguero[1], Manuel Yáñez[2,*], Otilia Mó[2], M. Merced Montero-Campillo[2]

[1] Instituto de Química Médica, IQM-CSIC. Juan de la Cierva, 3, E-28006 Madrid, Spain

[2] Departamento de Química, Módulo 13, Facultad de Ciencias and Institute of Advanced Chemical Sciences (IadChem), Universidad Autónoma de Madrid, Campus de Excelencia UAM-CSIC, Cantoblanco, 28049-Madrid, Spain

Index:

Pg. S2-17 Electronic energy (hartree) and Optimized geometry (Å, °) at MP2/aug-cc-pVTZ//aug-cc-pVTZ-PP computational level

Pg. S18 Table S1. Energy decomposition analysis components (kJ mol^{-1})

NH_3 complexes

F_2:NH_3	MP2= -255.75468851 NIMAG= 0<br>F<br>F,1,r1<br>X,2,1.,1,90.<br>N,2,rxb,3,90.,1,180.,0<br>H,4,r3,2,a3,3,0.,0<br>H,4,r3,2,a3,3,120.,0<br>H,4,r3,2,a3,3,-120.,0<br><br>r1=1.41629674<br>rxb=2.59357104<br>r3=1.01226183<br>a3=111.93157218
FCl:NH_3	MP2= -615.84120762 NIMAG= 0<br>F<br>Cl,1,r1<br>X,2,1.,1,90.<br>N,2,rxb,3,90.,1,180.,0<br>H,4,r3,2,a3,3,0.,0<br>H,4,r3,2,a3,3,120.,0<br>H,4,r3,2,a3,3,-120.,0<br><br>r1=1.714061<br>rxb=2.233069<br>r3=1.01226772<br>a3=110.1552828
Cl_2:NH_3	MP2= -975.85634482 NIMAG= 0<br>Cl<br>Cl,1,r1<br>X,2,1.,1,90.<br>N,2,rxb,3,90.,1,180.,0<br>H,4,r3,2,a3,3,0.,0<br>H,4,r3,2,a3,3,120.,0<br>H,4,r3,2,a3,3,-120.,0<br><br>r1=2.03365915<br>rxb=2.59183518<br>r3=1.01243456<br>a3=111.37679814
FBr:NH_3	MP2= -2728.85378351 NIMAG= 0<br>F<br>Br,1,r1<br>X,2,1.,1,90.<br>N,2,rxb,3,90.,1,180.,0<br>H,4,r3,2,a3,3,0.,0

	H,4,r3,2,a3,3,120.,0<br>H,4,r3,2,a3,3,-120.,0<br><br>r1=1.8287939<br>rxb=2.29289921<br>r3=1.0125857<br>a3=110.26325641
ClBr:NH_3	MP2= -3088.86007270 NIMAG= 0<br>Cl<br>Br,1,r1<br>X,2,1.,1,90.<br>N,2,rxb,3,90.,1,180.,0<br>H,4,r3,2,a3,3,0.,0<br>H,4,r3,2,a3,3,120.,0<br>H,4,r3,2,a3,3,-120.,0<br><br>r1=2.20281796<br>rxb=2.46906228<br>r3=1.0126311<br>a3=110.7595784
Br_2:NH_3	MP2= -5201.85272142 NIMAG= 0<br>Br<br>Br,1,r1<br>X,2,1.,1,90.<br>N,2,rxb,3,90.,1,180.,0<br>H,4,r3,2,a3,3,0.,0<br>H,4,r3,2,a3,3,120.,0<br>H,4,r3,2,a3,3,-120.,0<br><br>r1=2.33234293<br>rxb=2.53805106<br>r3=1.01252703<br>a3=110.8961989
FI:NH_3	MP2= -451.06713097 NIMAG= 0<br>F<br>I,1,r1<br>X,2,1.,1,90.<br>N,2,rxb,3,90.,1,180.,0<br>H,4,r3,2,a3,3,0.,0<br>H,4,r3,2,a3,3,120.,0<br>H,4,r3,2,a3,3,-120.,0<br><br>r1=1.97539948<br>rxb=2.44311965<br>r3=1.0131735<br>a3=110.78473124

$ClI:NH_3$	MP2= -811.06578565 NIMAG= 0<br>Cl<br>I,1,r1<br>X,2,1.,1,90.<br>N,2,rxb,3,90.,1,180.,0<br>H,4,r3,2,a3,3,0.,0<br>H,4,r3,2,a3,3,120.,0<br>H,4,r3,2,a3,3,-120.,0<br><br>r1=2.38960491<br>rxb=2.54041634<br>r3=1.01326895<br>a3=110.90712125
$BrI:NH_3$	MP2= -2924.05598844 NIMAG= 0<br>Br<br>I,1,r1<br>X,2,1.,1,90.<br>N,2,rxb,3,90.,1,180.,0<br>H,4,r3,2,a3,3,0.,0<br>H,4,r3,2,a3,3,120.,0<br>H,4,r3,2,a3,3,-120.,0<br><br>r1=2.52939367<br>rxb=2.58246094<br>r3=1.01320897<br>a3=110.94670559
$I_2:NH_3$	MP2= -646.24471190 NIMAG= 0<br>I<br>I,1,r1<br>X,2,1.,1,90.<br>N,2,rxb,3,90.,1,180.,0<br>H,4,r3,2,a3,3,0.,0<br>H,4,r3,2,a3,3,120.,0<br>H,4,r3,2,a3,3,-120.,0<br><br>r1=2.71689564<br>rxb=2.67161761<br>r3=1.01309125<br>a3=111.09038372
$FAt:NH_3$	MP2= -417.81071407 NIMAG= 0<br>F<br>At,1,r1<br>X,2,1.,1,90.<br>N,2,rxb,3,90.,1,180.,0<br>H,4,r3,2,a3,3,0.,0

	H,4,r3,2,a3,3,120.,0<br>H,4,r3,2,a3,3,-120.,0<br><br>r1=2.06201468<br>rxb=2.49591989<br>r3=1.0132176<br>a3=111.14241716
ClAt:NH_3	MP2= -777.81017922 NIMAG= 0<br>Cl<br>At,1,r1<br>X,2,1.,1,90.<br>N,2,rxb,3,90.,1,180.,0<br>H,4,r3,2,a3,3,0.,0<br>H,4,r3,2,a3,3,120.,0<br>H,4,r3,2,a3,3,-120.,0<br><br>r1=2.47934246<br>rxb=2.55399297<br>r3=1.01329783<br>a3=111.12041075
BrAt:NH_3	MP2= -2890.79993583 NIMAG= 0<br>Br<br>At,1,r1<br>X,2,1.,1,90.<br>N,2,rxb,3,90.,1,180.,0<br>H,4,r3,2,a3,3,0.,0<br>H,4,r3,2,a3,3,120.,0<br>H,4,r3,2,a3,3,-120.,0<br><br>r1=2.62032461<br>rxb=2.58210475<br>r3=1.01328385<br>a3=111.10874489
IAt:NH_3	MP2= -612.98783842 NIMAG= 0<br>I<br>At,1,r1<br>X,2,1.,1,90.<br>N,2,rxb,3,90.,1,180.,0<br>H,4,r3,2,a3,3,0.,0<br>H,4,r3,2,a3,3,120.,0<br>H,4,r3,2,a3,3,-120.,0<br><br>r1=2.81171215<br>rxb=2.63959257<br>r3=1.0132651<br>a3=111.15892892

At_2:NH_3	MP2= -579.72196274 NIMAG= 0<br>At<br>At,1,r1<br>X,2,1.,1,90.<br>N,2,rxb,3,90.,1,180.,0<br>H,4,r3,2,a3,3,0.,0<br>H,4,r3,2,a3,3,120.,0<br>H,4,r3,2,a3,3,-120.,0<br><br>r1=2.88906976<br>rxb=2.66734219<br>r3=1.01313515<br>a3=111.14259045

PH$_3$ complexes

F_2:PH_3	MP2= -541.95432363 NIMAG= 0<br>F<br>F,1,r1<br>X,2,1.,1,90.<br>P,2,rxb,3,90.,1,180.,0<br>H,4,r3,2,a3,3,0.,0<br>H,4,r3,2,a3,3,120.,0<br>H,4,r3,2,a3,3,-120.,0<br><br>r1=1.41188267<br>rxb=3.04815812<br>r3=1.41184501<br>a3=122.39260167
FCl:PH_3	MP2= -902.04251903 NIMAG= 0<br>F<br>Cl,1,r1<br>X,2,1.,1,90.<br>P,2,rxb,3,90.,1,180.,0<br>H,4,r3,2,a3,3,0.,0<br>H,4,r3,2,a3,3,120.,0<br>H,4,r3,2,a3,3,-120.,0<br><br>r1=1.8508506<br>rxb=2.18284465<br>r3=1.40140984<br>a3=116.7154972
Cl_2:PH_3	MP2= -1262.05385212 NIMAG= 0<br>Cl<br>Cl,1,r1<br>X,2,1.,1,90.<br>P,2,rxb,3,90.,1,180.,0<br>H,4,r3,2,a3,3,0.,0<br>H,4,r3,2,a3,3,120.,0<br>H,4,r3,2,a3,3,-120.,0<br><br>r1=2.02461019<br>rxb=3.05029103<br>r3=1.41031988<br>a3=121.64861689
FBr:PH_3	MP2= -3015.05287625 NIMAG= 0<br>F<br>Br,1,r1<br>X,2,1.,1,90.<br>P,2,rxb,3,90.,1,180.,0<br>H,4,r3,2,a3,3,0.,0

	H,4,r3,2,a3,3,120.,0<br>H,4,r3,2,a3,3,-120.,0<br><br>r1=1.89534983<br>rxb=2.38864529<br>r3=1.40237018<br>a3=117.6810174
ClBr:PH_3	MP2= -3375.05674853 NIMAG= 0<br>Cl<br>Br,1,r1<br>X,2,1.,1,90.<br>P,2,rxb,3,90.,1,180.,0<br>H,4,r3,2,a3,3,0.,0<br>H,4,r3,2,a3,3,120.,0<br>H,4,r3,2,a3,3,-120.,0<br><br>r1=2.24482976<br>rxb=2.65905645<br>r3=1.40588044<br>a3=119.37317054
Br_2:PH_3	MP2= -5488.04964841 NIMAG= 0<br>Br<br>Br,1,r1<br>X,2,1.,1,90.<br>P,2,rxb,3,90.,1,180.,0<br>H,4,r3,2,a3,3,0.,0<br>H,4,r3,2,a3,3,120.,0<br>H,4,r3,2,a3,3,-120.,0<br><br>r1=2.34486864<br>rxb=2.83570903<br>r3=1.40790653<br>a3=120.40984932
FI:PH_3	MP2= -737.26368739 NIMAG= 0<br>F<br>I,1,r1<br>X,2,1.,1,90.<br>P,2,rxb,3,90.,1,180.,0<br>H,4,r3,2,a3,3,0.,0<br>H,4,r3,2,a3,3,120.,0<br>H,4,r3,2,a3,3,-120.,0<br><br>r1=2.0024694<br>rxb=2.64173201<br>r3=1.40348205<br>a3=118.36858381

ClI:PH_3	MP2= -1097.26137326 NIMAG= 0<br>Cl<br>I,1,r1<br>X,2,1.,1,90.<br>P,2,rxb,3,90.,1,180.,0<br>H,4,r3,2,a3,3,0.,0<br>H,4,r3,2,a3,3,120.,0<br>H,4,r3,2,a3,3,-120.,0<br><br>r1=2.41462645<br>rxb=2.79240764<br>r3=1.40509443<br>a3=119.0753141
BrI:PH_3	MP2= -3210.25168113 NIMAG= 0<br>Br<br>I,1,r1<br>X,2,1.,1,90.<br>P,2,rxb,3,90.,1,180.,0<br>H,4,r3,2,a3,3,0.,0<br>H,4,r3,2,a3,3,120.,0<br>H,4,r3,2,a3,3,-120.,0<br><br>r1=2.54693265<br>rxb=2.86831914<br>r3=1.40610025<br>a3=119.54504004
I_2:PH_3	MP2= -932.44090194 NIMAG= 0<br>I<br>I,1,r1<br>X,2,1.,1,90.<br>P,2,rxb,3,90.,1,180.,0<br>H,4,r3,2,a3,3,0.,0<br>H,4,r3,2,a3,3,120.,0<br>H,4,r3,2,a3,3,-120.,0<br><br>r1=2.72004391<br>rxb=3.03258038<br>r3=1.40801602<br>a3=120.46472462
FAt:PH_3	MP2= -704.00753200 NIMAG= 0<br>F<br>At,1,r1<br>X,2,1.,1,90.<br>P,2,rxb,3,90.,1,180.,0<br>H,4,r3,2,a3,3,0.,0

	H,4,r3,2,a3,3,120.,0<br>H,4,r3,2,a3,3,-120.,0<br><br>r1=2.08297794<br>rxb=2.72125484<br>r3=1.40337976<br>a3=118.6125101
ClAt:PH_3	MP2= -1064.00631541 NIMAG= 0<br>Cl<br>At,1,r1<br>X,2,1.,1,90.<br>P,2,rxb,3,90.,1,180.,0<br>H,4,r3,2,a3,3,0.,0<br>H,4,r3,2,a3,3,120.,0<br>H,4,r3,2,a3,3,-120.,0<br><br>r1=2.50500692<br>rxb=2.80203753<br>r3=1.40421859<br>a3=118.897352
BrAt:PH_3	MP2= -3176.99603607 NIMAG= 0<br>Br<br>At,1,r1<br>X,2,1.,1,90.<br>P,2,rxb,3,90.,1,180.,0<br>H,4,r3,2,a3,3,0.,0<br>H,4,r3,2,a3,3,120.,0<br>H,4,r3,2,a3,3,-120.,0<br><br>r1=2.64275519<br>rxb=2.84474805<br>r3=1.4047981<br>a3=119.11654331
IAt:PH_3	MP2= -899.18397783 NIMAG= 0<br>I<br>At,1,r1<br>X,2,1.,1,90.<br>P,2,rxb,3,90.,1,180.,0<br>H,4,r3,2,a3,3,0.,0<br>H,4,r3,2,a3,3,120.,0<br>H,4,r3,2,a3,3,-120.,0<br><br>r1=2.82612986<br>rxb=2.93854893<br>r3=1.4060983<br>a3=119.67501528

At_2:PH_3	MP2= -865.91846576 NIMAG= 0<br>At<br>At,1,r1<br>X,2,1.,1,90.<br>P,2,rxb,3,90.,1,180.,0<br>H,4,r3,2,a3,3,0.,0<br>H,4,r3,2,a3,3,120.,0<br>H,4,r3,2,a3,3,-120.,0<br><br>r1=2.90003455<br>rxb=2.98061664<br>r3=1.40675757<br>a3=119.96788314

H_2O complexes

F_2:OH_2	MP2= -275.62217342 NIMAG= 0<br>F,1.7820075601,-0.065417344,0.0037161016<br>F,0.3753698684,-0.046653826,-0.0007681207<br>O,-2.2730710311,-0.039011243,-0.0005131851<br>H,-2.7244939109,0.3430606585,0.7577294241<br>H,-2.7451988187,0.3141355977,-0.76016422
FCl:OH_2	MP2= -635.69975104 NIMAG= 0<br>F,1.8521796224,-0.0296887564,-0.0022938264<br>Cl,0.196781258,-0.0653780035,0.0013504885<br>O,-2.3192804161,-0.1208031406,0.0062590909<br>H,-2.6572455843,0.375295106,0.7593350408<br>H,-2.6578212121,0.3466886378,-0.7646507939
Cl_2:OH_2	MP2= -995.72110781 NIMAG= 0<br>Cl,2.3016950541,-0.0554474843,-0.0027285506<br>Cl,0.2936565321,-0.0753519428,0.0032892135<br>O,-2.4806867579,-0.0945003294,0.0057299512<br>H,-2.8476874047,0.3822398791,0.7567788716<br>H,-2.8523637558,0.3491737207,-0.7630694858
FBr:OH_2	MP2= -2748.70882508 NIMAG= 0<br>F,1.9356368997,-0.0245365147,-0.006288334<br>Br,0.15579271,-0.0628219894,-0.0007798509<br>O,-2.3367130402,-0.1141351773,0.0058435232<br>H,-2.6682875381,0.3752122383,0.7672050712<br>H,-2.6712806901,0.3651909569,-0.760569223
ClBr:OH_2	MP2= -3108.72089915 NIMAG= 0<br>Cl,2.3739348901,-0.0558293231,0.0003775627<br>Br,0.2194543278,-0.0724313884,0.0008917601<br>O,-2.4782408765,-0.0928297748,0.0054111865<br>H,-2.8477744455,0.3802414747,0.7583239798<br>H,-2.852760228,0.3469628547,-0.7650044894
Br_2:OH_2	MP2= -5221.71484739 NIMAG= 0<br>Br,1.7024568281,0.0105979409,0.<br>Br,-0.5888553341,-0.0271340903,0.<br>O,-3.3454027973,-0.0637603509,0.<br>H,-3.7211181886,0.3903657864,0.7611839187<br>H,-3.7211181886,0.3903657864,-0.7611839187
FI:OH_2	MP2= -470.92093841 NIMAG= 0<br>F,1.3111392883,0.0281571908,0.0008483773<br>I,-0.6294772576,-0.0208785988,-0.0012439677<br>O,-3.2135434419,-0.0791019745,-0.0047553244<br>H,-3.5730567482,0.371773677,0.7679093453<br>H,-3.5698997413,0.3983884603,-0.7627584306

ClI:OH_2	MP2= -830.92355086 NIMAG= 0<br>Cl,1.7293875042,0.0108158308,0.0002069162<br>I,-0.6133871021,-0.0281673054,-0.000350912<br>O,-3.3428817573,-0.0610230296,-0.0003737808<br>H,-3.7187159147,0.3897164277,0.763829946<br>H,-3.7180583036,0.3924005743,-0.7633121694
BrI:OH_2	MP2= -2943.91496486 NIMAG= 0<br>Br,1.8879674147,0.01652205,0.<br>I,-0.5961780936,-0.0182544098,0.<br>O,-3.376957398,-0.0493743516,0.<br>H,-3.7640993293,0.3934546622,0.7628307035<br>H,-3.7640993293,0.3934546622,-0.7628307035
I_2:OH_2	MP2= -666.10586837 NIMAG= 0<br>I,1.70846828,0.01097688,0.<br>I,-0.969147,-0.01917171,0.<br>O,-3.83751255,-0.0421243,0.<br>H,-4.24128177,0.38515185,0.76294573<br>H,-4.24128177,0.38515185,-0.76294573
FAt:OH_2	MP2= -437.66319359 NIMAG= 0<br>F,-0.0055303921,0.,0.007516526<br>At,0.0041550477,0.,2.0377527348<br>O,0.0112029375,0.,4.6302931144<br>H,-0.4456363264,0.7661123261,4.9961391075<br>H,-0.4456363264,-0.7661123261,4.9961391075
ClAt:OH_2	MP2= -797.66532815 NIMAG= 0<br>Cl,0.0169720274,0.000198235,-0.3832688185<br>At,0.0086112566,-0.0003079908,2.0506997614<br>O,-0.0060279334,-0.0005104676,4.7396585872<br>H,-0.4518949298,0.7651465012,5.1190843447<br>H,-0.4554037146,-0.7645262778,5.1182622977
BrAt:OH_2	MP2= -2910.65609811 NIMAG= 0<br>Br,2.448342455,0.0282642996,-0.0000464014<br>At,-0.1266189474,-0.0147277109,0.0000068895<br>O,-2.8549329458,-0.0494568084,-0.0001934526<br>H,-3.2421563773,0.3911052603,0.7644123153<br>H,-3.2418532102,0.3924448103,-0.7641793508
IAt:OH_2	MP2= -632.84594802 NIMAG= 0<br>I,2.6460665415,0.0212284956,0.0019771178<br>At,-0.1233391982,-0.0157382121,-0.0036707801<br>O,-2.9221336596,-0.0424601799,-0.0076746952<br>H,-3.3225027755,0.3655467713,0.7677497254<br>H,-3.3215214136,0.410278263,-0.7583813679

At_2:OH_2	MP2= -599.58085909 NIMAG= 0<br>At,2.7313017684,0.0176322406,0.0007223929<br>At,-0.1207682583,-0.0170975203,-0.0021933986<br>O,-2.9484380545,-0.0402727839,-0.0023460509<br>H,-3.3483755405,0.3833008498,0.7648887373<br>H,-3.3494489949,0.3973789442,-0.7610716807

SH_2 complexes

F_2:SH_2	MP2= -598.20185351 NIMAG= 0<br>F,2.3465860899,-0.0970474283,-0.0031218846<br>F,0.9360221066,-0.1000931094,-0.0018230594<br>S,-2.0848601342,-0.064286329,-0.0074580254<br>H,-2.1575055425,0.8449477902,0.9693603632<br>H,-2.1347492141,0.8749267978,-0.9569573938
FCl:SH_2	MP2= -958.28047629 NIMAG= 0<br>F,2.4020228994,-0.088594387,0.0006158197<br>Cl,0.7204608602,-0.0824188569,-0.002370275<br>S,-2.0002617204,-0.0697616937,-0.007640341<br>H,-2.110654673,0.836384689,0.9701498014<br>H,-2.1060740605,0.86283797,-0.9607550051
Cl_2:SH_2	MP2= -1318.30106828 NIMAG= 0<br>Cl,2.8588522505,-0.1331798498,0.0003879121<br>Cl,0.8424911141,-0.1229298729,-0.0027420954<br>S,-2.2568463683,-0.0460101839,-0.0071315622<br>H,-2.2719337245,0.8678934841,0.9686760906<br>H,-2.2670699661,0.892674144,-0.9591903451
FBr:SH_2	MP2= -3071.29056972 NIMAG= 0<br>F,2.5114962025,-0.1144970602,-0.0072398755<br>Br,0.7039439966,-0.0907446236,-0.0010828145<br>S,-2.0156822122,-0.0614065273,0.0088532164<br>H,-2.1379783493,0.8657464593,0.9660765384<br>H,-2.147063211,0.8452753496,-0.9666070648
ClBr:SH_2	MP2= -3431.30142776 NIMAG= 0<br>Cl,2.921495485,-0.1369548113,-0.00018186<br>Br,0.7478239578,-0.1136045323,-0.0030778664<br>S,-2.2233964808,-0.047026982,-0.0062078515<br>H,-2.2710330905,0.8665608337,0.9696867875<br>H,-2.2693965658,0.8894732135,-0.9602192095
Br_2:SH_2	MP2= -5544.29539551 NIMAG= 0<br>Br,3.0536637724,-0.1414478613,0.<br>Br,0.74597777,-0.126907049,0.<br>S,-2.2977933969,-0.0515978189,0.<br>H,-2.3121814674,0.8747439663,0.9645999635<br>H,-2.3121814674,0.8747439663,-0.9645999635
FI:SH_2	MP2= -793.50249266 NIMAG= 0<br>F,2.7243085819,-0.1302345597,0.0024646952<br>I,0.7663215517,-0.0953299969,-0.0001944106<br>S,-2.1096164144,-0.**0575167088**,-0.0041248448<br>H,-2.2518536249,0.8519116337,0.9676998897<br>H,-2.2494177647,0.862966842,-0.9658453294

ClI:SH_2	MP2= -1153.50435317 NIMAG= 0<br>Cl,3.1164796193,-0.1635470338,0.0003283312<br>I,0.7547242513,-0.1181582813,-0.0003543589<br>S,-2.2762491692,-0.0444380099,-0.0009454657<br>H,-2.356992964,0.8766405686,0.9665199719<br>H,-2.3574714527,0.8795973386,-0.9655484785
BrI:SH_2	MP2= -3266.49571655 NIMAG= 0<br>Br,-2.2007479255,-0.0255665782,0.<br>I,0.300995711,0.0292400571,0.<br>S,3.3874781126,0.067093077,0.<br>H,3.4815191493,-0.8544824696,0.9655509459<br>H,3.4815191493,-0.8544824696,-0.9655509459
I_2:SH_2	MP2= -988.68642794 NIMAG= 0<br>I,-2.006171861,-0.0204434888,0.<br>I,0.6860531565,0.0377950126,0.<br>S,3.8760886657,0.0517643736,0.<br>H,3.9282026753,-0.8733445718,0.9649429922<br>H,3.9282026753,-0.8733445718,-0.9649429922
FAt:SH_2	MP2= -760.24521160 NIMAG= 0<br>F,-0.1056672739,-0.0018372596,0.1796599863<br>At,0.0269249352,-0.0003036523,2.2217748403<br>S,0.2346900725,0.0019638189,5.1177747685<br>H,-0.6626751804,0.9672733826,5.3525855065<br>H,-0.6586090767,-0.9670962897,5.3525838371
ClAt:SH_2	MP2= -1120.24691136 NIMAG= 0<br>Cl,2.7141062567,0.031193775,0.0025611526<br>At,0.2598335446,-0.0233583281,-0.0063159051<br>S,-2.7422242959,-0.0914881411,-0.0177313842<br>H,-2.8979865276,0.7908479002,0.9767124187<br>H,-2.8909481764,0.852268098,-0.955226282
BrAt:SH_2	MP2= -3233.23760801 NIMAG= 0<br>Br,2.8433456028,0.0246252955,0.0025444436<br>At,0.2479532241,-0.0315873289,-0.0065888362<br>S,-2.7922865385,-0.0879264809,-0.0176491344<br>H,-2.9279417614,0.7978415407,0.9764127259<br>H,-2.9212629377,0.8588647167,-0.9547191989
IAt:SH_2	MP2= -955.42719864 NIMAG= 0<br>I,3.0288786838,0.019314927,0.0018000694<br>At,0.2408535614,-0.0414957745,-0.0068904183<br>S,-2.8750236081,-0.0867176618,-0.0175758686<br>H,-2.9806618138,0.8028037331,0.9762008951<br>H,-2.9719502535,0.8643502918,-0.9535346776

At_2:SH_2	MP2= -922.16213346 NIMAG= 0<br>At,3.1050105379,0.0151040543,0.0009309249<br>At,0.2368764679,-0.0473774903,-0.0053093606<br>S,-2.9092516748,-0.0841042946,-0.0130605991<br>H,-2.9954105566,0.8153771413,0.9736666703<br>H,-2.9879681155,0.8615858898,-0.9562276355

Table S1. Energy decomposition analysis components (kJ mol^{-1})

	Base = NH_3			OH_2			PH_3			SH_2		
X-Y	E_{Pauli}	V_{elst}	E_{orb}	E_{Pauli}	V_{elst}	E_{orb}	E_{Pauli}	V_{elst}	E_{orb}	E_{Pauli}	V_{elst}	E_{orb}
F_2	27.0	-17.9	-27.2	11.8	-7.6	-7.4	18.3	-10.1	-18.8	17.2	-9.6	-19.1
FCl	250.2	-159.2	-163.7	60.3	-45.5	-40.8	749.2	-254.6	-889.1	120.8	-70.3	-93.0
Cl_2	85.0	-59.4	-56.9	27.4	-21.4	-16.0	55.4	-33.4	-40.3	41.6	-26.1	-31.1
FBr	279.8	-194.1	-164.3	91.1	-69.6	-55.7	523.1	-297.5	-325.3	165.5	-101.1	-114.9
ClBr	172.3	-120.9	-100.9	48.9	-38.8	-28.0	249.9	-145.6	-155.1	83.7	-53.1	-57.8
Br_2	142.6	-99.7	-83.5	41.7	-32.4	-23.0	150.4	-89.3	-95.0	69.5	-43.9	-47.4
FI	283.0	-207.8	-149.6	113.6	-87.4	-61.4	399.6	-243.6	-234.5	171.3	-109.0	-109.1
ClI	225.3	-162.4	-116.7	76.0	-59.2	-39.2	275.3	-167.3	-158.5	117.2	-75.0	-72.3
BrI	203.9	-145.5	-104.9	66.2	-51.1	-33.3	226.4	-137.4	-129.4	103.0	-65.7	-62.6
I_2	162.3	-115.0	-82.5	52.1	-39.9	-24.9	145.8	-88.7	-82.3	80.1	-50.9	-46.9
FAt	306.2	-229.2	-145.8	142.8	-107.7	-68.3	403.3	-253.7	-218.5	203.0	-130.2	-115.1
ClAt	274.3	-199.1	-126.5	112.1	-83.4	-51.2	340.1	-210.5	-176.7	163.3	-103.7	-88.2
BrAt	259.0	-185.5	-118.2	102.1	-75.1	-45.8	308.5	-189.7	-157.9	150.8	-95.1	-79.8
IAt	226.9	-159.9	-101.6	84.9	-61.7	-36.7	244.7	-148.9	-121.7	126.8	-79.3	-64.7
At_2	213.8	-148.6	-93.1	79.6	-56.9	-33.0	221.5	-133.5	-106.3	119.4	-73.8	-58.2
